# Energy Expenditure, a New Tool for Monitoring Surgical Stress in Colorectal Oncological Patients: A Prospective, Monocentric Study

**DOI:** 10.7759/cureus.56822

**Published:** 2024-03-24

**Authors:** Robert Ivascu, Madalina Dutu, Dan Corneci, Cornelia Nitipir

**Affiliations:** 1 Anesthesia and Critical Care, Dr. Carol Davila University Emergency Central Military Hospital, Bucharest, ROU; 2 Anesthesia and Critical Care, Carol Davila University of Medicine and Pharmacy, Bucharest, ROU; 3 Oncology, Elias University Emergency Hospital, Bucharest, ROU; 4 Oncology, Carol Davila University of Medicine and Pharmacy, Bucharest, ROU

**Keywords:** oncological outcome, indirect calorimetry, cortisol dynamic, energy expenditure, surgical stress response

## Abstract

Background: Surgical stress response in colorectal surgery consists of a neurohormonal and an immunological response and influences oncological outcomes. The intensity of surgical trauma influences mortality, morbidity, and metastasis’ occurrence in colorectal neoplasia. Energy expenditure (EE) stands for the body’s energy consumed to keep its homeostasis and can be either calculated or measured by direct or indirect calorimetry.

Aim: The present study attempted to evaluate surgical stress response using EE measurement and compare it to the postoperative cortisol dynamic.

Methods: A prospective, monocentric study was conducted over a period of one year in the Anesthesiology Department including 21 patients from whom serum cortisol values were collected in the preoperative period and on the first postoperative day, and EE was measured and recorded every 15 minutes throughout surgery using the indirect calorimetry method. The study compared EE values’ dynamic registered 30 minutes after intubation and 30 minutes before extubating (after abdominal closure) to cortisol perioperative dynamic.

Results: We enrolled 21 patients and 84 measurements were recorded, 42 probes of serum cortisol and 42 measurements of EE. The mean value of the first measurement of serum cortisol was 13.60±3.6 µg and the second was 16.21±6.52 µg. The average value of the first EE recording was 1273.9±278 kcal and 1463.4±398.2 kcal of the second recording. The bivariate analysis performed showed a good correlation between cortisol variation and EE's variation (Spearman coefficient=0.666, p<0.001, CI=0.285, 0.865). In nine cases (42.85%), cortisol value at 24 hours reached the baseline or below the baselines preoperative value. In eight cases (38.09%), patients’ EE at the end of the surgery was lower than that recorded at the beginning of the surgery.

Conclusions: Intraoperative EE variation correlated well with cortisol perioperative dynamic and stood out in this study as a valuable and accessible predictor of surgical stress in colorectal surgery.

## Introduction

Colorectal cancer is the second cause of mortality among oncological pathologies. In 2020, more than two million cases were newly diagnosed, and the number of new cases is expected to increase by more than 50% until 2040 [1]. The key element in colorectal cancer treatment is surgery, both laparoscopic and open surgery causing a controlled tissue trauma. Surgical stress response consists of a neurohormonal response and an immunological response. Up to now, biomarkers such as vasopressin, adrenocorticotropic hormones or cortisol, insulin, and glucose have been used to evaluate the neurohormonal response and interleukin 10, interleukin 8, interleukin 6, interleukin 1β, tumor necrosis factor, and ferritin have been used for immunological response [2-6]. Although they represent a systemic evaluation of surgical stress response, these biomarkers are not used in regular medical practice. Considering the fact that surgical stress influences oncological prognosis and that the intensity of surgical trauma influences mortality, morbidity, and metastasis’ occurrence in colorectal neoplasia, it is worthwhile to investigate whether a non-invasive method could determine the intensity of surgical stress and the possibility to use it on a daily basis in the operating room [7-9].

Energy expenditure (EE) stands for the body’s energy consumed to keep its homeostasis. Oxygen is the main element in the energy production process, being involved in the oxidative phosphorylation cycle, carbon dioxide and water being the main resulting products [10]. EE can be either calculated or measured by direct or indirect calorimetry. Quantification through indirect calorimetry is based on breath-by-breath measurement of oxygen consumption and carbon dioxide production [11]. The present study attempted to evaluate surgical stress response using EE measurement and compare it to the postoperative cortisol dynamic. To the authors' knowledge, it is the first time that indirect calorimetry has been used in the surgery room for this purpose.

## Materials and methods

Patients and study design

This is a cross-sectional, prospective, single-center study conducted over a period of one year, from February 2023 to February 2024, in the Anesthesiology Department of Dr. Carol Davila University Emergency Central Military Hospital, Bucharest, aiming to find a new tool for surgical stress response evaluation. Thus, we compare the serum cortisol variation in the perioperative period with the intraoperative EE variation.

The study was approved by the Ethics Committee of the University Emergency Central Military Hospital Dr. Carol Davila (no: 3272/02 May 2022). All the procedures performed in this study followed the ethical standards of the Declaration of Helsinki.

Only patients with colorectal oncological disease, confirmed by histopathological analysis, scheduled for elective surgical intervention with an oncological curative visa were eligible. They must also meet the following criteria: age between 18 and 80 years and American Society of Anesthesiologists (https://www.asahq.org) class I, II, or III.

The first exclusion criterion was an allergy to anesthetic substances. Patients with stage IV oncological pathology were also excluded because we considered that the surgical intervention did not have a curative purpose. Taking into account that atrial fibrillation and neurological comorbidities (Alzheimer's and Parkinson's disease) interfere with the results provided by the device used to individualize anesthetic management, they represented other exclusion criteria. The carbon dioxide used in intraoperative interventions influences the indirect calorimetry values; therefore, this surgical technique was excluded.

Anesthetic management 

According to the American Society of Anesthesiologists Standard, anesthesia monitoring includes electrocardiogram, non-invasive blood pressure, pulse oximetry, capnography, and temperature. General anesthesia’s depth was monitored using entropy (Monitor Carescape B650, soft version 24.4, General Electric Healthcare, Helsinki, Finland) and analgesia was evaluated with surgical pleth index (SPI) (Monitor Carescape B650, soft version 24.4, General Electric HealthCare, Helsinki, Finland). To measure and monitor EE, the EsCOVX module (General Electric HealthCare, Helsinki, Finland) was used. Anesthesia induction used propofol (titrated for an entropy value: response entropy and state entropy 40-60), fentanyl 3 mg/kg, rocuronium 0.6 mg/kg, and lidocaine 2 mg/kg. Anesthesia was maintained with sevoflurane and fentanyl, hypnosis depth was monitored with entropy (response and state entropy: target values 40-60), and analgesia intensity with SPI (target values 20-50). Lidocaine 1.5 mg/kg/h and ketamine 2 5mg/kg/h in continuous infusion have also been administered post-induction. Postoperative nausea and vomiting prophylaxis have been performed.

Data recording

Cortisol values were collected in the preoperative period (Mc1) and on the first postoperative day, 20 hours after the end of the surgery (Mc2), using the chemiluminescent method with microparticles (Abbot analyzer). EE has been measured and recorded every 15 minutes throughout surgery using the indirect calorimetry method (EsCOVX). We compared EE values dynamic registered at 30 minutes after intubation (Mee1) and 30 minutes before extubating (after abdominal closure) (Mee2) to blood cortisol level variation. Cortisol and EE variation have been calculated using the mathematical formula: ((Mc2-Mc1)/Mc1)*100 for serum cortisol and ((Mee2-Mee1)/Mee1)*100 for EE. When measured variation was above zero, a positive trend was considered and values below zero represented a negative trend for both cortisol and EE. Hemodynamic and respiratory parameters have also been recorded (CarescapeB650 monitor, software version 24.4, General Electric Healthcare, Helsinki, Finland).

Statistics

No data were found in the specialized literature about the intraoperative use of indirect calorimetry so, for this paper, which can be considered a pilot study, the sample size could not be estimated before starting the study. Continuous variables would be expressed as mean and standard deviation if the variables were normally distributed and as median and interquartile range if the variables were non-normally distributed. Categorical variables were reported as numbers and as frequency (%). Ryan Joiner's statistical test was used to check normality for quantitative variables (p>0.05). Bivariate analysis using Spearman's rho coefficient was conducted to investigate the correlation between the perioperative variation of EE and the variation of cortisol. In all tests, a P-value lower than 0.05 was considered statistically significant. The statistical analysis was performed with Minitab software for Windows version 21 (Minitab LLC, State College, Pennsylvania, USA).

## Results

Upon applying inclusion and exclusion criteria, 10 patients were excluded because of incorrect cortisol collection. The study enrolled 21 patients. Table 1 presents the demographic characteristics of patients as well as data from the perioperative period. 

**Table 1:** Demographic characteristics and perioperative data ^1^Median (interquartile range: 25-75th percentile) ^2^percentages ^3 ^mean ± standard deviation ASA, American Society of Anesthesiologists; NRS 2002, Nutrition Risk Screening 2002; SPI, surgical pleth index

Parameter	
Demographics	
Age^1^, years	63 (55-73)
Gender	
Women	7 (30%)
Men	14 (60%)
BMI^3^, kg/m^2 ^	26.06±4.55
Weight^3^, kg	75.5±15.22
Medical History	
Arterial hypertension^2^	11 (52.38%)
Diabetes mellitus^2^	3 (14.28%)
Heart failure^2^	2 (9.52%)
Chronic kidney disease^2^	2 (9.52%)
Myocardial infarction^2^	2 (9.52%)
Peripheral vascular disease^2^	3 (14.28%)
Surgery Characteristics	
Duration of surgery^3^,minutes	265.7±91.4
Intraoperative Monitoring	
Heart rate^1^, bpm	64 (58-75)
Systolic blood pressure^1^, mmHg	113 (99-131)
Diastolic blood pressure^1^, mmHg	67 (57-74)
Temperature^1^, Celsius degrees	36.1 (35.8-36.2)
Peripheral oxygen saturation^1^	98 (98-99)
Oxygen inspiratory fraction^1^	45 (40-50)
Response entropy^1^	49 (44-56)
State entropy^1^	44 (38-51)
SPI^1^	40 (27-58)
Preoperative Evaluation	
ASA^2^	
ASA II	11 (52.38%)
ASA III	10 (47.60%)
NRS 2002^2^	
Low risk	20 (95.23%)

The association between cortisol variation and energy expenditure

**Figure 1:**
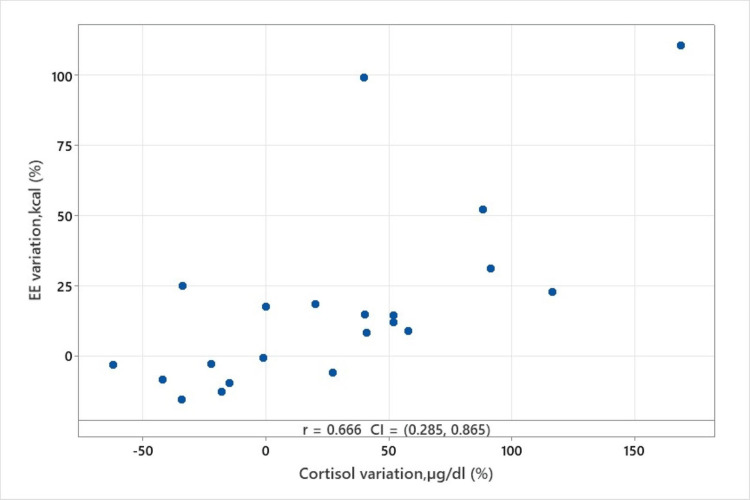
Cortisol and EE variation correlation, Spearman's rank correlation coefficient EE, energy expenditure

We compared cortisol and EE variation using the above-mentioned formula. Ryan Joiner's statistical test proved that our samples had a non-normal distribution (p<0.01) so the Spearman test (Rho) for correlation was applied (Figure 1).

The bivariate analysis performed shows a good correlation between cortisol variation and EE’s variation (Spearman coefficient=0.666, p<0.001, CI=0.285, 0.865).

Cortisol and energy expenditure variation trend

In 21 patients, 84 measurements were recorded, 42 probes of serum cortisol and 42 measurements of EE. The mean value of the first measurement of serum cortisol was 13.60±3.6 µg/dL and the second was 16.21±6.52 µg/dl. The average value of the first EE recording was 1273.9±278 kcal and 1463.4±398.2 kcal for the second recording. In nine cases (42.85%), the postoperative cortisol value registered at 24 hours reached the baseline or below baselines preoperative value, meaning a negative trend variation. In eight cases (38.09%), patients’ EE at the end of the surgery was lower than the value recorded at the beginning of the surgery, meaning also a negative variation trend (Table 2).

**Table 2:** EE and serum cortisol trend EE variation=((Mee2-Mee1)/Mee1)*100; serum cortisol variation=((Mc2-Mc1)/Mc1)*100; positive trend if variation >0, negative trend if variation <0 EE, energy expenditure

Parameter	Number of patients (n, %)
EE variation	
Positive trend	13 (61.91%)
Negative trend	8 (38.09%)
Serum cortisol variation	
Positive trend	12 (57.14%)
Negative trend	9 (42.85%)

**Figure 2:**
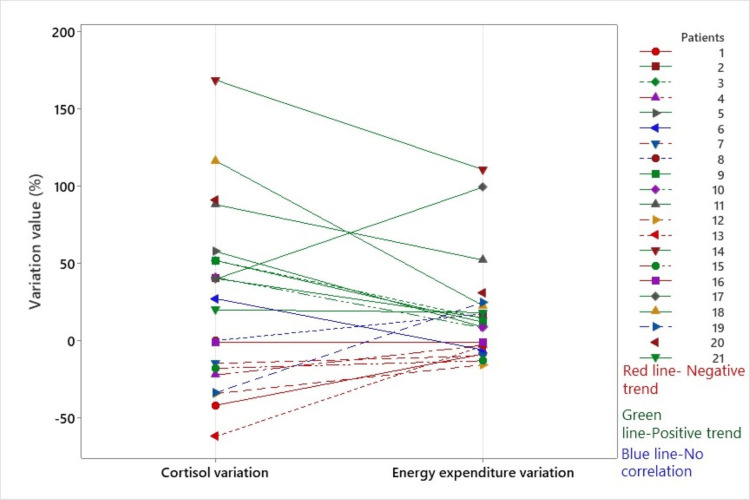
Cortisol and EE trends (positive, negative, and non-concordant trends) EE, energy expenditure

For every patient, we compared cortisol and EE variation using a line plot graph (Figure 2). We noticed a concordant positive trend (green lines) for 11 patients, a concordant negative trend (red lines) for seven patients, and in three cases, a discordant trend was registered (blue lines).

## Discussion

Surgery causes trauma to the body that tries to maintain its homeostasis and facilitate postoperative healing by producing a systemic response that generates hormonal and metabolic changes [12].

The early postoperative outcome is influenced by the size of surgical trauma. Changes produced in the hemato-immunological axis generate Th1 lymphocyte cell suppression, with a consecutive Th1/Th2 lymphocyte ratio reduction, favoring the appearance of infectious complications [13]. Sympatho-adrenomedullary axis (SAM) and the hypothalamic-pituitary adrenal-axis activation can be a trigger for the occurrence of cardiac, neurological, renal, hepatic, and coagulant dysfunction, thus increasing patients’ mortality and morbidity 30 days after surgery [14]. Long-term survival is also affected, and perioperative period changes create an environment favoring metastases’ occurrence [15,16]. Since 2003, Tsuchiya has demonstrated that high surgical stress in the perioperative period is associated with an increased rate of metastases [17]. This finding raises the question of whether surgical stress should be quantified in real-time and whether patients showing increased values should be monitored more closely concerning the oncological outcome.

Until now, the quantification of surgical stress has been studied only experimentally. Main biomarkers studied are ACTH, vasopressin, cortisol, growth hormone and insulin for neurohormonal response assessment and interleukin 1β, interleukin IL6, interleukin IL 8, interleukin IL 10, and interleukin 13 for hemato-immunolgic response. Among these, the most important one is cortisol. The amount of cortisol secreted depends on the magnitude of the surgery [18]. Because surgical stress is thought to have a prognostic value for oncological patients, our aim was to demonstrate that EE could be an available tool to quantify intraoperative metabolic disturbances, mirroring cortisol perioperative dynamic.

Measuring EE can be easily done by indirect calorimetry, which relies on oxygen consumed and carbon dioxide produced from respiratory gases [19]. The gas module uses side-stream gas sampling and has an infrared sensor for carbon dioxide measurement and a paramagnetic sensor for oxygen assessment. Three formulas can decide EE. (1) Oxygen consumption (VO_2_)=Mvinsp*FiO_2_-Mvexp*FeO_2_ (where Mvinsp=inhaled ventilator minute, Mvexp=exhaled ventilator minute, FiO_2_=inspiratory oxygen fraction, and FeO_2_=expiratory fraction 02), (2) carbon dioxide production (VCO_2_)=MVexp*FeCO_2_-Mvinsp*FiCO_2 _(FeCo_2_=fraction of carbon dioxide exhaled and FiCo_2_=fraction of carbon dioxide inhaled), and (3) EE=5.5 Vo_2 _(mL/min)+1.76 VCO_2 _(mL/min)-2UN (g/day).

From the mathematical model expression, we understand the importance of oxygen fraction delivered to the intubated patient. When ventilating a patient with a FiO_2_ between 0.4 and 0.8, in his study Ferreruela noticed no statistical change in EE [20]. The recommendation found in the literature is to take with caution data at FiO_2_ higher than 0.6, with measurements’ accuracy decreasing with the increase in the amount of O_2_ delivered [21]. In our study, the median value of oxygen fraction was 40 (mean: 40, first quartile (Q1): 40, third quartile (Q3): 50). It should be also mentioned that in patients on continuous renal replacement therapy, EE value could also be affected [22,23].

Temperature has a major influence on all chemical and biochemical reactions that take place in a living organism. Thus, the temperature increase is associated with a higher rate of metabolism, and its decrease is accompanied by cellular functions’ downturn. The recommended temperature value during the intraoperative period is 35.5-37 degrees Celsius. The median temperature value in our study was 36.1°Celsius (Q1: 35.8 and Q3: 36.2). Entropy is a useful tool for anesthetic depth monitoring as it processes electroencephalogram and frontal electromyogram waves and transforms them into numerical values [24]. Values recorded both for RE (response entropy) (median value: 49, Q1: 44-Q3: 56) and for SE (state entropy) (median 44, Q1: 38-Q3: 51) were within the limits indicated by the literature [25]. SPI stands for the balance between the degree of nociceptors’ activation and administered analgesia, using the plethysmography arterioles signals [26]. We have obtained a median value of 40 (Q1: 27-Q3: 58). The level of anesthetic depth, intraoperative pain, and temperature can independently influence cellular metabolism. Because all these parameters proved to be within recommended limits, we could state that surgical trauma and cellular response were the main determinants of metabolic rate.

Statistical analyses revealed a good correlation between the cortisol variation and EE variation (Spearman coefficient Rh0=0.666, p<0.05). However, what drew our attention was that not all patients had a positive trend of cortisol variation. In nine cases (42.85%), the cortisol value measured 20 hours after the end of the surgery returned to the initial value or even below the baseline. The same negative trend of EE variation was concordant for 38.09% of patients. This physiological fluctuation can have several explanations. First, surgical stress was very well addressed by anesthesia; despite individualization of anesthetic management (both hypnosis and analgesia being measured objectively in real-time), anesthetics were administered according to the patient's needs and not at fixed intervals. Another explanation could be the time of day when the intervention was performed; Kwon's study revealed that the percentage of patients whose cortisol level returned to baseline was much higher when surgery was performed in the afternoon [27]. This is an interesting topic, and we will address it in the following studies.

Until now, EE has been measured only in the preoperative and postoperative periods, not intraoperatively. Thus, Silva measured EE preoperatively, and then on day three or five postoperatively, his results showed an increase in EE value in only 33% of patients [28]. Ukleja in 2016 determined EE preoperatively and postoperatively finding no significant differences [29]. Chen tried to measure postoperative EE and compared it to values calculated using the predictive equation, without notable results [30].

Although there is a good correlation between cortisol and EE, intraoperative EE variation cannot be interpreted independently in a clinical context. Further studies are needed to establish a reference value for EE variation to help clinicians detect the possibility of complicated outcomes earlier in oncological patients.

This study has its drawbacks. A limitation would be the small number of patients admitted to the study. Although statistically relevant (p<0.05), the result must be confirmed by further studies, especially since it has been the first time, as far as the authors know, that tool was used to evaluate surgical stress response. Considering fluctuating cortisol’s metabolism, its measurement at closer intervals and over a longer period would be proper. It should also be mentioned that although all patients included in the study were not known to have hypothalamic-pituitary-adrenal axis pathologies, no further investigations were done to confirm this.

## Conclusions

Intraoperative EE variation correlated well with cortisol perioperative dynamic in oncological patients. EE stood out in this study as a non-invasive reliable and accessible predictor of surgical stress in colorectal surgery.
